# Deep Learning-Based IoT System for Remote Monitoring and Early Detection of Health Issues in Real-Time

**DOI:** 10.3390/s23115204

**Published:** 2023-05-30

**Authors:** Md. Reazul Islam, Md. Mohsin Kabir, Muhammad Firoz Mridha, Sultan Alfarhood, Mejdl Safran, Dunren Che

**Affiliations:** 1 Department of Computer Science and Engineering, Bangladesh University of Business and Technology, Dhaka 1216, Bangladesh; mailforreazul@gmail.com (M.R.I.); mdmkabi@gmail.com (M.M.K.); 2 Department of Computer Science, American International University-Bangladesh, Dhaka 1229, Bangladesh; firoz.mridha@aiub.edu; 3 Department of Computer Science, College of Computer and Information Sciences, King Saud University, P.O. Box 51178, Riyadh 11543, Saudi Arabia; mejdl@ksu.edu.sa; 4 School of Computing, Southern Illinois University, Carbondale, IL 62901, USA; dche@cs.siu.edu

**Keywords:** convolutional neural network, Internet of Things, deep learning, medical IoT, sensor

## Abstract

With an aging population and increased chronic diseases, remote health monitoring has become critical to improving patient care and reducing healthcare costs. The Internet of Things (IoT) has recently drawn much interest as a potential remote health monitoring remedy. IoT-based systems can gather and analyze a wide range of physiological data, including blood oxygen levels, heart rates, body temperatures, and ECG signals, and then provide real-time feedback to medical professionals so they may take appropriate action. This paper proposes an IoT-based system for remote monitoring and early detection of health problems in home clinical settings. The system comprises three sensor types: MAX30100 for measuring blood oxygen level and heart rate; AD8232 ECG sensor module for ECG signal data; and MLX90614 non-contact infrared sensor for body temperature. The collected data is transmitted to a server using the MQTT protocol. A pre-trained deep learning model based on a convolutional neural network with an attention layer is used on the server to classify potential diseases. The system can detect five different categories of heartbeats: Normal Beat, Supraventricular premature beat, Premature ventricular contraction, Fusion of ventricular, and Unclassifiable beat from ECG sensor data and fever or non-fever from body temperature. Furthermore, the system provides a report on the patient’s heart rate and oxygen level, indicating whether they are within normal ranges or not. The system automatically connects the user to the nearest doctor for further diagnosis if any critical abnormalities are detected.

## 1. Introduction

The healthcare industry has experienced a significant evolution in recent years and has evolved into a prominent benefactor to revenue and employment [1]. Traditional methods of diagnosing diseases and abnormalities in the human body required physical analysis in a hospital setting, which often necessitated extended hospital stays for patients during their treatment period. This approach increased healthcare costs and strained healthcare facilities, particularly in rural and remote locations. However, technological advancements, including the use of miniaturized devices such as smartwatches, have allowed for the diagnosis of various diseases and health monitoring, transforming a hospital-centric healthcare system into a patient-centric one [2]. Moreover, the aging population and the prevalence of chronic diseases have created tremendous pressure on the healthcare industry, leading to the emergence of remote health monitoring as a promising solution for improving patient care, reducing healthcare costs, and minimizing hospital visits [3]. Remote health monitoring involves collecting various physiological data, such as blood oxygen level, heart rate, body temperature, and ECG signals, which can be analyzed to identify potential health issues and provide real-time feedback to healthcare providers [4].

Integrating IoT and deep learning technologies has revolutionized medical systems in home environments by enabling remote health monitoring and early detection of health issues [5]. The use of IoT technology enables the collection of vast amounts of physiological data, such as blood oxygen level, heart rate, body temperature, and ECG signals, from wearable devices or sensors [6,7,8]. The collected data is transmitted to a remote server for analysis using deep learning algorithms to detect patterns indicative of various health issues. Deep learning algorithms, such as convolutional neural networks (CNNs), can analyze large amounts of data, and automatically learn to extract informative features that are indicative of specific health issues [9]. This eliminates the need for manual feature extraction, which can be time-consuming and error-prone. In addition to CNNs, deep learning algorithms can also be enhanced with attention layers that highlight the most informative features in the input data, improving the model’s performance. Attention layers enable the deep learning model to focus on specific features of the input data that are most relevant to the classification task [10]. This improves accuracy and reduces computational complexity, making it suitable for deployment in resource-constrained environments such as home healthcare systems [11].

This paper proposes an IoT-based system for remote monitoring and early detection of health issues in home clinical settings. The system utilizes three types of sensors, including the MAX30100, AD8232 ECG sensor module, and MLX90614 Non-contact Infrared body Temperature Sensor, to collect physiological data from the human body. The collected data is then transmitted to a server using the MQTT protocol for analysis. At the server, we utilize a pre-trained deep learning model based on CNN with attention layers to classify probable diseases based on the collected physiological data. The CNN model is trained on a large dataset of physiological data and can identify patterns that are indicative of various health issues. The attention layer highlights the most informative features in the input data, enhancing the model’s performance. In addition, the system generates a report on the patient’s temperature, heart rate, and oxygen level, indicating whether they fall within normal ranges or not.

Combining IoT and deep learning technologies in medical systems for home environments has several advantages, including real-time monitoring, reduced hospital visits, and timely intervention, improving patient care and improving health outcomes [12]. The use of remote health monitoring systems can also provide patients with personalized feedback and advice, enabling them to take a more active role in their healthcare. Incorporating IoT and deep learning technologies in medical systems for home environments can transform the healthcare industry, enabling healthcare providers to provide more personalized care while reducing healthcare costs and improving health outcomes [13].

After conducting several experiments and analyses, this paper makes several significant contributions to the field of medical systems for home environments. The main contributions of this research can be outlined as follows:

- Development of a medical system for home environments that incorporates IoT and deep learning technologies to monitor health conditions such as arrhythmia, fever, heart rate, and oxygen levels.
- Apply a deep learning model based on CNN with attention layers to classify potential heart issues, including five types of arrhythmias: normal beat, supraventricular premature beat, premature ventricular contraction, fusion of ventricular, unclassifiable beat.
- Demonstration of the high accuracy and performance of the proposed deep learning model in identifying heart conditions achieving an accuracy of 0.982.
- Investigating the potential of integrating IoT and deep learning technologies in medical systems for home environments to provide real-time monitoring, timely intervention, and improved patient care while reducing healthcare costs and hospital visits.

The paper is structured as follows: Section 2 comprehensively reviews related work. Section 3 describes the methodology and materials used in the study, including IoT devices and sensors, data transmission and analysis, deep learning model design and implementation, and the proposed framework. Section 4 presents the analysis of the results, including data collection and preprocessing, evaluation metrics, training and testing of the deep learning model, and performance analysis. Section 5 provides an in-depth discussion of the study’s findings and limitations. Finally, Section 6 presents the conclusion and future directions for research in this field.

## 2. Related Work

IoT devices and sensors for remote health monitoring have been extensively explored in the literature [14,15,16,17,18,19]. These sensors can collect physiological data, which can be used for real-time health monitoring and disease diagnosis. Deep learning models have also been widely applied to remote health monitoring [20,21]. In particular, convolutional neural networks have shown promising results in classifying various health issues based on physiological data [22,23]. In addition, attention-based deep learning models have been proposed to improve the performance of health issue classification tasks [24]. Several studies have focused on designing and implementing deep learning models for remote health monitoring using IoT devices and sensors [25,26]. These studies have explored deep-learning architectures and evaluated their performance in detecting health issues.

Remote health monitoring has emerged as a promising solution for improving patient care and reducing healthcare costs. It involves collecting various physiological data that can be analyzed to identify potential health issues and provide real-time feedback to healthcare providers. Remote health monitoring has become increasingly popular with the advent of wearable devices and IoT technology, enabling continuous and non-invasive monitoring of physiological parameters. In the context of this paper, remote health monitoring is achieved through the use of IoT devices such as the MAX30100, AD8232 ECG sensor module, and the MLX90614 non-contact infrared body temperature sensor. These devices are used to collect physiological data from patients in a home clinical setting, which is then transmitted to a remote server for analysis. The collected data is analyzed using a deep learning model and simple thresholding to detect patterns indicative of various health issues, including five types of arrhythmias, fever, and normal conditions.

Remote health monitoring has several advantages over traditional hospital-centric healthcare systems. It enables patients to receive continuous care in the comfort of their homes, reducing the need for hospital visits and minimizing the risk of hospital-acquired infections. Remote health monitoring can also facilitate the early detection of health issues, enabling timely intervention and improving patient outcomes. Additionally, remote health monitoring can reduce healthcare costs by minimizing hospital visits and enabling more efficient use of healthcare resources.

Several related works have explored different aspects of heart disease detection and predictive modeling in healthcare. Tiwari et al. [27] developed the Smart Cardiovascular Disease Detection System (SCDDS), which integrates IoT sensors in a wearable device for early detection of heart disease. Their ensemble architecture, combining Convolutional Neural Networks (ConvNet) and ConvNet-LSTM, achieves a high accuracy of 98% in automatically detecting atrial fibrillation heartbeats through cloud-based deep learning architecture. Chadiya et al. [28] proposed a hybrid human-machine intelligence approach for creating predictive models in healthcare. By incorporating the expertise and reasoning of expert physicians and using quality-approved data, their study focused on predicting the course of Multiple Sclerosis (MS). The hybrid approach, which outperformed conventional methods, highlights the potential of integrating human and artificial intelligence to improve machine learning models and facilitate personalized clinical decision-making. Botros et al. [29] introduced two models, a CNN and an extended version with a Support Vector Machine (SVM) layer, for automatic detection of heart failure from electrocardiogram signals. These models achieved exceptional performance with an accuracy, sensitivity, and specificity of over 99% in detecting HF. Their proposed framework provides reliable references for doctors and enables real-time patient monitoring using portable devices.

Chandrasekhar et al. [30] present a study focused on enhancing the accuracy of heart disease prediction using machine learning algorithms. Six algorithms were employed: random forest, K-nearest neighbor, logistic regression, Naïve Bayes, gradient boosting, and the AdaBoost classifier. The models were evaluated using datasets from the Cleveland and IEEE Dataport databases. The logistic regression algorithm achieved the highest accuracy of 90.16% on the Cleveland dataset, while AdaBoost performed best on the IEEE Dataport dataset with 90% accuracy. Combining all six algorithms using a soft voting ensemble classifier improved the accuracy to 93.44% for the Cleveland dataset and 95% for the IEEE Dataport dataset.

Mirjalali et al. [31] reviewed various wearable sensors and their potential uses in remote health monitoring systems that can detect physiological and biochemical markers by estimating essential symptoms such as respiratory rate, body temperature, and blood oxygen level. The review focused on the latest advances in wearable sensor techniques that can be used to estimate crucial signals accurately, matching those of point-of-care tests, with an emphasis on the use of wearable sensors for assessing respiratory demeanor. The paper summarized strategies based on diverse materials and functional tools, emphasizing the potential use of wearable sensors towards noninvasive and premature diagnosis of many medical conditions, including COVID-19. The review pointed out the remaining difficulties and future possibilities in this emerging domain of remote medical monitoring, highlighted the need for further research to unlock the full potential of wearable sensors for remote health monitoring.

IoT and deep learning have appeared as promising technologies in the healthcare industry. Hammad et al. [32] proposed an approach for automatically detecting arrhythmia in IoT applications using CNN and the long-short-term (ConvLSTM) technique. The input ECG signals are transformed into 2D images and then classified using their proposed models, which address issues such as overfitting and working with multiple leads of ECG signals. The models were evaluated on several publicly available datasets, including MIT-BIH, PhysioNet 2016, and PhysioNet 2018, with overall accuracies of 0.97, 0.98, 0.94, and 0.91 achieved, respectively, on these datasets. In ref. [33], Nancy et al. utilized bidirectional long-short-term memory (Bi-LSTM) to monitor and predict heart disease risk. The system achieved an impressive accuracy of 0.98, a precision of 0.989, a sensitivity of 0.988, a specificity of 0.988, and an F-measure of 0.9886, outperforming existing intelligent heart condition prediction methods. The reported work emphasizes the importance of accurate and timely disease prediction to ensure preventative maintenance and earlier intervention for at-risk individuals. The integration of IoT and deep learning technologies in healthcare has the potential to revolutionize disease prediction and improve healthcare outcomes. Haq et al. [34] presented a prosperous brain tumor categorization technique utilizing deep learning strategies. An improved CNN architecture was employed to categorize brain tumors using brain magnetic resonance (MR) image data. Data augmentation and transfer learning techniques were incorporated to enhance the model’s classification performance. The proposed model outperformed the baseline models with high accuracy, indicating its potential for brain cancer diagnosis in IoT healthcare procedures.

Deep learning models have been increasingly used for health issue classification through the automatic extraction of relevant features from raw input data [35,36]. In particular, convolutional neural networks have shown great success in image-based classification tasks, while recurrent neural networks (RNNs) are better suited for sequence-based data [37]. Recently, hybrid models that combine CNNs and RNNs, such as convolutional long short-term memory networks, have emerged as powerful tools for processing image and sequence data processing [32]. These models have been applied to various health-related classification tasks, such as arrhythmia detection, disease diagnosis, and drug discovery. One of the major advantages of deep learning models is their ability to learn from large datasets, resulting in highly accurate classification models. Additionally, transfer learning and data augmentation techniques have been utilized to improve the performance of deep learning models, especially in cases where limited data is available [38]. Through their integration with IoT devices and cloud computing, deep learning models have the potential to revolutionize healthcare by enabling remote health monitoring and accurate diagnosis of diseases.

The related works reviewed above collectively deliver practical papers in this section that deliver practical insights into the possibility of leveraging IoT and deep learning technologies in personal healthcare systems, indicating a strong case and highlighting the necessity for continued investigation and development of remote mote medical monitoring systems in this field.

## 3. Methods and Materials

This section describes the methods and materials used in this research, including the IoT devices and sensors, data transmission and analysis, deep learning model design and implementation, and the proposed framework. These hardware were obtained from Ibrahim Electronics, a company located in Dhaka, Bangladesh. Various brands produce different components. The NodeMCU is manufactured by Espressif Systems in Shanghai, China. Medico Electrodes International Limited in Noida, India manufactures the AD8232 ECG sensor. Tianshui Huatian Sensor Co., Ltd in Tianshui, China manufactures the Max30100 and MLX90614 temperature sensors.

### 3.1. IoT Devices and Sensors

**Figure 1:**
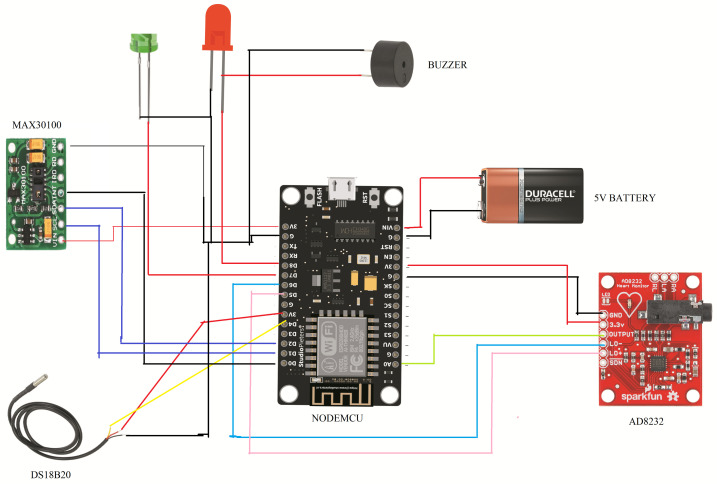
A schematic that incorporates all the elements and linkages in the circuit.

This study used three types of sensors: MAX30100, AD8232 ECG sensor module, and MLX90614 Non-contact Infrared body Temperature Sensor and one NodeMCU. The circuit diagram is presented in Figure 1.

NodeMCU: NodeMCU is an open-source firmware and development board designed to facilitate building IoT devices. The device is based on the ESP8266 Wi-Fi module, which is a low-cost Wi-Fi module with full TCP/IP stack capability. The NodeMCU board includes a USB interface for programming and powering the board and a voltage regulator to provide a stable 3.3 V power supply. The board includes 11 digital input/output pins, one analog input pin, and one UART (Universal Asynchronous Receiver-Transmitter) communication interface. The NodeMCU firmware is compatible with the Arduino IDE (Integrated Development Environment) and allows users to program the board using a variant of the C++ programming language. With the NodeMCU board, we can easily create connected devices that can send and receive data over Wi-Fi. The NodeMCU board was chosen because it is a development board specifically designed to build IoT devices. It is based on the ESP8266 Wi-Fi module, which provides a low-cost and efficient solution for wireless connectivity.

MAX30100: The MAX30100 is a high-sensitivity pulse oximeter and heart rate sensor module. It uses an integrated pulse oximetry and heart rate monitor sensor solution, including internal LEDs, photodetectors, and low-noise electronics with ambient light rejection. The sensor module can measure blood oxygen saturation level (SpO2) and heart rate (HR) with high accuracy and reliability. The sensor utilizes a red and infrared LED to illuminate the skin and measure the reflected light. The reflected light is then detected by a photodetector and processed to obtain SpO2 and HR values. The MAX30100 sensor module also includes an internal ambient light cancellation algorithm to improve accuracy and reduce the effects of ambient light on measurements. This sensor was selected because it incorporates LEDs, photodetectors, and low-noise electronics to measure blood oxygen saturation and heart rate accurately.

AD8232 ECG sensor: The AD8232 is a single-lead electrocardiogram (ECG) sensor designed to measure the heart’s electrical activity. It is a low-power, fully-integrated signal-conditioning block for biopotential measurements. The AD8232 ECG sensor is specifically designed for wearable applications where low power consumption and a small form factor are critical. The sensor can measure ECG signals with high accuracy and stability and can be used to detect arrhythmias, heart rate, and other cardiac-related parameters. We have employed the AD8232 ECG sensor because this sensor features an instrumentation amplifier, a right-leg drive amplifier, a lead-off detection circuit, and a comparator to capture and process the heart’s electrical activity.

MLX90614 Temperature Sensor: The MLX90614 is a non-contact infrared temperature sensor that measures the temperature of an object without the need for physical contact. The sensor uses an infrared-sensitive thermopile detector to measure the temperature of the target object. It converts the temperature into an electrical signal that can be read by a microcontroller. The sensor has a wide temperature measurement range from −70 °C to 380 °C. The MLX90614 has two sensing elements for ambient and object temperatures, respectively. It also includes a signal processing IC that provides a calibrated digital output for both temperature values over an I2C interface. This sensor was chosen because of the sensor’s small size, low power consumption, and non-contact measurement.

Table 1 presents the configurations and specifications of the components used in our IoT system.

### 3.2. Data Transmission and Analysis

MQTT (Message Queuing Telemetry Transport) is a lightweight and efficient protocol designed for Internet of Things (IoT) devices. It is used for reliable and low-latency communication between devices with constrained resources and limited bandwidth. In the context of this project, the sensor data was collected by the NodeMCU device and then transmitted to a remote server using the MQTT protocol. The NodeMCU device acted as an MQTT client, and the remote server acted as an MQTT broker. The NodeMCU device published the sensor data as messages on specific topics to the MQTT broker, who received them and forwarded them to the subscribed clients. In this case, the subscribed client was the server that received and stored the sensor data for further analysis and processing. The MQTT protocol uses a publish-subscribe model, where the publisher (NodeMCU device) sends messages to a specific topic, and the subscriber (server) receives messages from that topic. The protocol also allows for quality-of-service (QoS) levels to ensure reliable delivery of messages, even in unreliable network conditions.

Once the data is transmitted to the remote server, it undergoes several processing steps to extract meaningful information, which are explained in Section 4.

### 3.3. Deep Learning Model

The implementation of the CNN with the attention layer was carried out in Python using the Keras framework. A detailed description of the model architecture is presented in the subsequent section. The proposed model consists of three convolutional layers, an attention layer, and three fully connected layers.

#### 3.3.1. Convolutional Layers

The first convolutional layer has 64 filters with a kernel size of five and a ReLU activation function. The input shape is (186, 1), representing the length of the ECG signal and its channel. After each convolutional layer, the Batch Normalization layer is added to improve the model’s performance. The Max Pooling layer reduces the spatial dimensions of the output feature maps. The second convolutional layer has 128 filters with a kernel size of five and a ReLU activation function. The Max Pooling layer is again applied after this layer. The third convolutional layer has 256 filters with a kernel size of five and a ReLU activation function. The Max Pooling layer is applied for the last time after this layer.

Here, let *x* be the input signal, *W* be the weight matrix of the filter, *b* be the bias vector, and *f* be the activation function (in this case, ReLU). Then the output *y* of the convolutional layer is given by:

(1) y=f(x∗W+b)

where ∗ denotes the convolution operation.

Batch Normalization is applied after each convolutional layer to normalize the output and improve the model’s performance. Let *x* be the input, γ, and β be the learned scale and shift parameters, respectively. Then the output y of the Batch Normalization layer is given by:

(2) $$\mu_{B}=\frac{1}{m}\sum_{i=1}^{m}x_{i}$$

(3) $$\sigma_{B}^{2}=\frac{1}{m}\sum_{i=1}^{m}{\left(x_{i}-\mu_{B}\right)}^{2}$$

(4) $${\hat{x}}_{i}=\frac{x_{i}-\mu_{B}}{\sqrt{\sigma_{B}^{2}+\epsilon }}$$

(5) $$y_{i}=\gamma {\hat{x}}_{i}+\beta$$

where *m* is the batch size and ϵ is a small constant added for numerical stability.

Finally, the Max Pooling layer reduces the spatial dimensions of the output feature maps. Let *x* be the input and *s* be the pooling size. Then the output *y* of the Max Pooling layer is given by:

(6) $$y_{i,j}=\max_{p=1}^{s}\max_{q=1}^{s}x_{(i-1)s+p,(j-1)s+q}$$

where *i* and *j* are the indices of the output feature map.

#### 3.3.2. Attention Layer

The attention layer is added after the third convolutional layer to improve the model’s performance by focusing on the most informative regions of the input signal. The attention layer is defined as follows:

(7) q=tanh(xW+b)

where *q* is the attention score, *x* is the input, *W* is the weight matrix, and *b* is the bias vector. The softmax function is applied to *q* to obtain the attention weights, which are then multiplied with the input signal *x* to obtain the attended signal.

(8) a=softmax(q)

(9) $$attended_{signal}=a*x$$

#### 3.3.3. Fully Connected Layers

After the attention layer, the attended signal is flattened and passed through two fully connected layers. The first fully connected layer has 512 units with a ReLU activation function, followed by a Batch Normalization layer and a Dropout layer with a rate of 0.5. The second fully connected layer has 256 units with a ReLU activation function, followed by a Batch Normalization layer and a Dropout layer with a rate of 0.5.

Let *x* be the input, *W* be the weight matrix, *b* be the bias vector, and *f* be the activation function (in this case, ReLU). Then the output y of the fully connected layer is given by:

(10) y=f(xW+b)

Then the Batch normalization technique is used to normalize each layer’s inputs, which helps speed up the training process and reduce the model’s sensitivity to the initialization of the weights.

Then the Dropout layer prevents overfitting by randomly dropping out some of the neurons during training. Let *x* be the input and *p* be the dropout rate. Then the output *y* of the Dropout layer is given by:

(11) $$y_{i}=\left\{\begin{array}{cc} x_{i} & \mathrm{with}\;\mathrm{probability}\;1-p\\ 0 & \mathrm{with}\;\mathrm{probability}\;p\; \end{array}\right.$$

where *i* is the index of the neuron.

#### 3.3.4. Output Layer

The Softmax activation function is used in the output layer to normalize the network’s output into a probability distribution. Let *z* be the previous layer’s output and *s* be the softmax function. Then the output *y* of the Softmax layer is given by:

(12) $$y_{i}=s\left(z_{i}\right)=\frac{e^{z_{i}}}{\sum_{j=1}^{K}e^{z_{j}}}$$

where *i* is the neuron index, *K* is the number of output neurons, and *e* is Euler’s number.

In summary, the proposed CNN model with an attention mechanism enhances its ability to focus on relevant input signal features, improving its classification task performance.

### 3.4. Proposed IoT and Deep Learning-Based Framework

The proposed framework involves the installation of three sensors—the AD8232 ECG sensor, the MAX30100 Pulse Oximeter, and the MLX90614 Non-contact Infrared body Temperature Sensor—on the human body to collect various types of data, including ECG signals, heart rate, blood oxygen level, and body temperature. The collected data is then transmitted to a remote server through the NodeMCU using the MQTT protocol for further processing.

The data is passed to the proposed deep learning model, which classifies the data based on its type. Specifically, the ECG signals and heart rate data are analyzed to detect five types of heart problems (normal beat, supraventricular premature beat, premature ventricular contraction, fusion of ventricular and unclassifiable beat). In contrast, the body temperature data is analyzed to determine if a fever is present. Moreover, the system generates a report indicating whether the patient’s temperature, heart rate, and oxygen level are within normal limits or not. The system then decides whether the detected condition is alarming or not. If the situation is dangerous, the system connects with the nearest doctor for further diagnosis and treatment.

**Figure 2:**
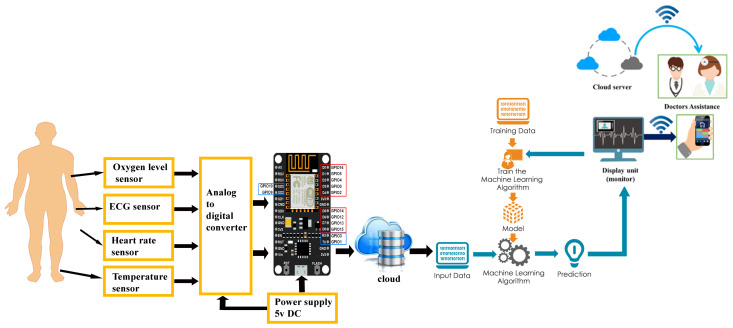
Proposed framework for remote health monitoring and early detection using IoT sensors and deep learning algorithms.

Overall, the proposed framework utilizes a combination of IoT sensors and deep learning algorithms to enable remote health monitoring and early detection of health issues. By leveraging the power of deep learning, the system can accurately analyze vast amounts of data and make informed decisions about patient health, leading to better healthcare outcomes and improved patient care. The proposed framework is illustrated in Figure 2.

## 4. Experimental Results

In this section, we will explain the results obtained in our study. This will include details on the data collection and processing methods used as well as an overview of the accuracy metrics utilized to evaluate the performance of our models. Finally, we will analyze the obtained results.

### 4.1. Data Collection and Preprocessing

This study used the publicly available ECG Heartbeat Categorization Dataset - MIT-BIH Arrhythmia Database [39] to train and evaluate our deep learning-based IoT system. The dataset contains ECG recordings from various patients and includes five different categories of heartbeats: normal beat, supraventricular premature beat, premature ventricular contraction, fusion of ventricular, and unclassifiable beat. We used the AD8232 ECG sensor module to collect the patients’ ECG signals. The module is a low-power, single-lead, and heart-rate monitor analog front end for all types of portable applications. The sensor module gains 1000 and outputs an analog voltage signal proportional to the heart’s electrical activity.

We collected ECG signals from 22 patients with various heart conditions. The signals were recorded at a sampling frequency of 250 Hz and a resolution of 10 bits. Each recording was 10 s long and contained 2500 samples. Before feeding the ECG signals into the pre-trained CNN model, we performed several preprocessing steps to ensure the data was compatible with the model’s input requirements. First, we applied a bandpass filter to remove any noise outside the frequency range of 0.5 to 100 Hz. We then resampled the signals to a frequency of 125 Hz to reduce the computational cost of the model. Finally, we normalized the signals by subtracting the mean and dividing them by the standard deviation.

Despite being primarily trained and evaluated on the ECG Heartbeat Categorization Dataset, we also tested the model with real-time data collected in our lab.

### 4.2. Evaluation Metrices

We used several standard evaluation metrics to evaluate the system’s performance, including accuracy, precision, recall, and F1-score.

The accuracy is the ratio of correctly classified samples to the total number of samples in the dataset:

(13) $$\mathrm{Accuracy}=\frac{\mathrm{TP}+\mathrm{TN}}{\mathrm{TP}+\mathrm{FP}+\mathrm{TN}+\mathrm{FN}}$$

where TP is the number of true positive samples, TN is the number of true negative samples, FP is the number of false positive samples, and FN is the number of false negative samples.

Precision is the ratio of true positive samples to the total number of positive samples predicted by the model:

(14) $$\mathrm{Precision}=\frac{\mathrm{TP}}{\mathrm{TP}+\mathrm{FP}}$$

The recall is the ratio of true positive samples to the total number of positive samples in the dataset:

(15) $$\mathrm{Recall}=\frac{\mathrm{TP}}{\mathrm{TP}+\mathrm{FN}}$$

The F1-score is the harmonic mean of precision and recall and provides a balanced measure of the model’s performance:

(16) $$F1\text{-}\mathrm{score}=2\frac{\mathrm{Precision}*\mathrm{Recall}}{\mathrm{Precision}+\mathrm{Recall}}$$

We calculated these metrics for each category of heartbeats in the ECG Heartbeat Categorization Dataset and the model’s overall performance.

### 4.3. Experimental Setup and Hyperparameter Optimization

In the experimental setup and validation, we conducted our experiments using Keras and TensorFlow within a virtual environment. The setup consisted of an RTX3070 GPU, a Ryzen 7 processor, and 16 GB of RAM. To optimize our model’s performance, we employed the grid search technique to find the optimal combinations of hyperparameters, including the number of filters, kernel size, dropout rate, and learning rate. The best results obtained from this optimization process are presented in the paper.

After multiple adjustments, we established a six-layer model consisting of the following layers: input layer, Convolutional layer 1, Convolutional layer 2, attention layer, fully connected layer, and output layer. We experimented with various filter sizes and settled on 64, 128, and 256 combinations. For the dense layer, we employed 512 and 256 neurons. Both the filter sizes and the number of neurons were determined using the grid search technique. Additionally, we tested different dropout values (0.2, 0.5, and 0.8) and found 0.5 to be the most optimal. Similarly, we fine-tuned the learning rate with values such as 0.1, 0.01, and 0.001, ultimately selecting 0.001 as the optimal value. In the final experiment, we used a batch size of 512 and trained the model for 25 epochs. Table 2 displays the hyperparameters fine-tuned using the grid search technique.

### 4.4. Results Analysis

The experiment trained a CNN with an attention layer model to categorize electrocardiogram heartbeat signals into five classes. The model achieved an overall accuracy of 0.982, indicating that it performs well in distinguishing between different types of heartbeats.

**Figure 3:**
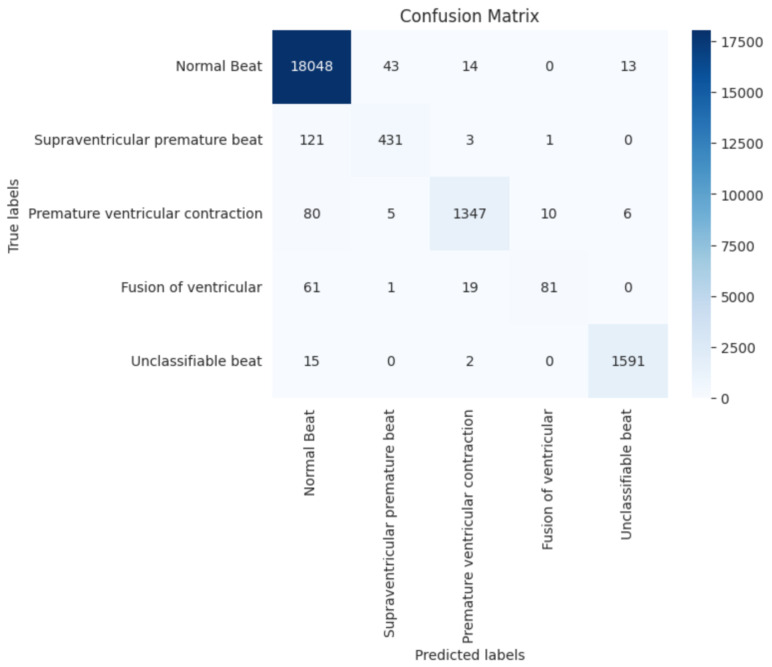
Confusion Matrix for the proposed model Performance.

Figure 3 shows the confusion matrix, and we can see that the model has a high number of true positives and true negatives for most classes. However, there are some instances where the model makes errors. For example, there are 121 instances where the model incorrectly identifies ventricular ectopic beats as normal heartbeats (class 0). Similarly, there are 61 instances where the model incorrectly identifies fusion heartbeats as normal heartbeats. These errors could potentially have implications for medical diagnosis and treatment.

Table 3 shows each class’s precision, recall, and f1-score; we can see that the model performs exceptionally well for class 0, with a precision of 0.98 and a recall of 1.00. This suggests that the model is very good at identifying normal heartbeats. For class 1, which represents ventricular ectopic beats, the model achieves a precision of 0.90 and a recall of 0.78. While the precision is relatively high, the recall is somewhat lower, which indicates that the model sometimes struggles to identify ventricular ectopic beats correctly.

For class 2, which represents supraventricular ectopic beats, the model achieves a precision of 0.97 and a recall of 0.93. This suggests that the model is very good at identifying supraventricular ectopic beats. For class 3, which represents fusion heartbeats, the model achieves a precision of 0.88 and a recall of 0.50. While the precision is relatively high, the recall is quite low, indicating that the model sometimes struggles to identify fusion heartbeats correctly. Finally, for class 4, which represents unknown beats, the model achieves a precision of 0.99 and a recall of 0.99, indicating that the model is very good at identifying these beats.

The results presented in Table 4 demonstrate the effectiveness of the proposed method for arrhythmia detection, which uses a CNN with attention layers. The proposed method achieved an accuracy of 0.982 and an F1-score of 0.980, which are higher than most of the other methods listed in the table.

Compared with other deep learning approaches, such as deep CNNs and CNNs with LSTM layers, the proposed method achieved higher accuracy and F1-score, indicating its superior performance for arrhythmia detection. For instance, Chen et al. [40] reported an accuracy of 0.992 and an F1-score of 0.908 for a CNN with LSTM layers, while Yıldırım et al. [41] reported an accuracy of 0.913 and an F1-score of 0.851 for a deep CNN. The proposed method outperformed other approaches using advanced techniques, such as support vector machines and genetic algorithms. For example, Plawiak et al. [42] reported an accuracy of 0.910 and an F1-score of 0.894 for an SVM-based neural system, while Hammad et al. [43] achieved an accuracy of 0.980 and an F1-score of 0.959 using a combination of deep neural networks, genetic algorithms, and k-nearest neighbors. In addition, the proposed method achieved comparable performance to some of the most recent state-of-the-art approaches for arrhythmia detection, such as Kim et al. [44] and Mohebbian et al. [32]. Kim et al. [44] reported an accuracy of 0.992 and an F1-score of 0.916 for a ResNet with biLSTM layers, while Mohebbian et al. [32] achieved an accuracy of 0.980 using semi-supervised active transfer learning. Overall, the results demonstrate that the proposed method, which uses a CNN with attention layers, is a highly effective approach for arrhythmia detection, outperforming most state-of-the-art methods in terms of accuracy and F1-score.

**Table 4:** Performance comparison of state-of-the-art methods for arrhythmia detection in terms of accuracy and F1-score.

Ref.	Year	Methodology	Performance
Achariya et al. [45]	2017	Deep CNN	Acc = 0.945 F1_score = 0.715
Plawiak et al. [42]	2018	SVM based Neural System	Acc = 0.910 F1_score = 0.894
Yıldırım et al. [41]	2018	Deep CNN	Acc = 0.913 F1_score = 0.851
Chen et al. [40]	2020	CNN+LSTM	Acc = 0.992 F1_score = 0.908
Yao et al. [46]	2020	ATI-CNN	Acc = 0.812
Zhou and Tan [47]	2020	CNN	Acc = 0.975
Hammad et al. [43]	2020	DNN + GA + KNN	Acc = 0.980 F1_score = 0.959
Kim et al. [44]	2022	ResNet + biLSTM	Acc = 0.992 F1_score = 0.916
Mohebbian et al. [32]	2023	Semi-supervised active transfer learning	Acc = 0.980
**Proposed method**	**2023**	**CNN with attention layers**	**Acc = 0.982** **F1_score = 0.980**

The proposed system uses heart rate as one of the key indicators for checking the heart condition of the individual being monitored. We obtain heart rate data by age, which allows us to consider the normal range of heart rates for each age group. For example, newborns aged 0 to 1 month typically have a heart rate ranging from 70 to 190 beats per minute (bpm), while infants aged 1 to 11 months have a heart rate of 80 to 160 bpm. As children grow, their heart rate tends to decrease, with heart rates for children aged 10 years and older, as well as adults and seniors, falling within the normal range of 60 to 100 bpm. By comparing an individual’s current heart rate with the normal range for their age group, our system can detect any abnormal heart rates and notify the appropriate healthcare provider for further evaluation and treatment if necessary.

Furthermore, we have used a simple thresholding approach with a fixed threshold value of 100.4 °F to classify cases as either fever or non-fever. This method involves classifying all cases above the threshold as fever and those below the threshold as non-fever. This approach is often used in medical diagnosis, where a specific temperature threshold is used to classify patients as having a fever. While simple thresholding can be effective in some cases, it may not be suitable for all situations, and more sophisticated methods may be required.

This system also closely monitors patients’ oxygen levels. The typical range for oxygen saturation is between 95 and 100 percent. If the levels are lower than this, it may indicate several medical conditions such as lung disease, heart disease, anemia, or carbon monoxide poisoning. Conversely, if the oxygen levels are excessively high, it can result in cellular damage and inflammation. Hence, it is essential to monitor oxygen levels closely, and our system provides real-time updates to doctors to ensure prompt action.

**Figure 4:**
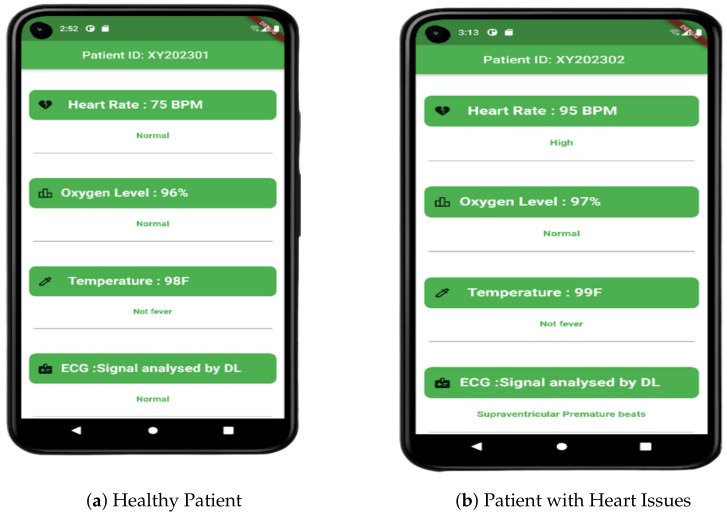
The patient data, including heart rate, oxygen level, temperature, and ECG signal, undergoes real-time analysis.

The health conditions of two individuals are depicted in Figure 4 using data collected in our laboratory. The first individual shows normal readings for all parameters, while the second individual has been diagnosed with supraventricular premature beats, a heart condition. The data from both individuals are fed into our application from the cloud, and we utilize a thresholding method based on heart rate, oxygen level, and temperature to monitor their condition. In addition, we employ our Convolutional Neural Network with attention layers to analyze the ECG data to classify the heart condition.

In conclusion, the proposed framework has been designed to detect and classify various human abnormalities, such as severe heart conditions and fever, and connect the nearest medical facility for further diagnosis. The framework employs a deep learning architecture consisting of a CNN with attention layers to classify heart conditions. This CNN model is trained on ECG signals and can classify five types of heart conditions: normal beat, supraventricular premature beat, premature ventricular contraction, Fusion of ventricular, and unclassifiable beat. Additionally, the framework incorporates a simple thresholding approach for classifying fever conditions. Furthermore, the framework monitors the oxygen level of the patient, which can provide valuable information about other medical conditions. By combining these various features, the proposed framework has the potential to provide rapid and accurate diagnoses, which could greatly improve patient outcomes. Overall, the findings of this study suggest that the proposed framework could be a promising tool for diagnosing and monitoring various medical conditions.

## 5. Discussion and Future Research

Home environment health monitoring is a promising solution to improve healthcare outcomes and reduce healthcare costs. However, several challenges must be addressed to ensure the successful implementation of these systems. One of the primary challenges is the accuracy and reliability of the data collected from the sensors. The data collected from the sensors can be affected by several factors, such as environmental conditions, device malfunctions, and user errors, which can result in inaccurate and unreliable data. Another challenge is the privacy and security of the collected data. The collected data contains sensitive and personal information about the users, and any breach of this data can have severe consequences for their privacy and security.

To address these challenges, this research proposes a novel framework for remote health monitoring using IoT and deep learning technologies. The proposed framework utilizes three sensors, including the AD8232 ECG sensor, MAX30100 pulse oximeter sensor, and MLX90614 non-contact infrared body temperature sensor, to collect various physiological data such as ECG signals, heart rate, oxygen level, and body temperature. The collected data is transmitted to a remote server using the MQTT protocol for analysis using deep learning algorithms. The proposed deep learning model is designed to classify the collected data into different categories based on the data type. The model utilizes a CNN with attention layers to improve the accuracy and performance of the model.

The proposed framework has several advantages, including real-time monitoring, reduced hospital visits, and timely intervention, which can improve patient care and improve health outcomes. The integration of IoT and deep learning technologies in healthcare has the potential to revolutionize disease prediction and improve healthcare outcomes. The proposed framework can also address the challenges of the accuracy and reliability of the data collected from the sensors. The deep learning algorithms used in the framework can analyze vast amounts of data and eliminate the need for manual feature extraction, improving the accuracy and reliability of the data collected from the sensors.

The proposed framework also addresses the privacy and security challenges of the collected data. The collected data is transmitted to a remote server using the MQTT protocol, which provides secure and encrypted data transmission. The collected data is stored on a remote server, which is accessible only to authorized personnel, ensuring the privacy and security of the collected data.

In the proposed model, attention layers are utilized within a CNN architecture to enhance its performance in heart disease classification. Attention layers are crucial when incorporated into CNN architectures for heart disease classification tasks. Attention mechanisms allow the network to focus on important features or regions within the input data, enhancing the model’s ability to make accurate predictions and improving its interpretability. In the context of heart disease classification, attention layers can provide valuable insights into the relevant regions or patterns within electrocardiogram signals. By incorporating attention layers, the CNN can learn to attend to specific ECG segments that are highly indicative of heart diseases or specific waveform characteristics. This attention-based approach helps the model capture fine-grained details and local dependencies within the ECG signal, improving its ability to discriminate between different heart disease classes.

Furthermore, to ensure convenient usage for both patients and doctors, we have developed a mobile application using Flutter. The app allows easy sign-up and sign-in processes for both parties, storing their information securely on the server. As patients input their measurements, the system automatically processes the data, providing the services described in this research.

In conclusion, this research proposes a novel framework for remote health monitoring using IoT and deep learning technologies. The proposed framework addresses the challenges of accuracy and reliability of the data collected from the sensors, privacy, and security of the collected data. The proposed deep learning model achieved an accuracy of 0.982, outperforming existing smart heart disease prediction systems. The integration of IoT and deep learning technologies in healthcare has the potential to revolutionize disease prediction and improve healthcare outcomes.

Further exploration of this domain can be carried out, with potential research directions including:

- Integration of more sensors and data sources: While our proposed framework utilizes multiple sensors for data collection and analysis, many other sensors and data sources can be integrated to provide a more comprehensive picture of an individual’s health status. For example, integrating sensors that measure blood pressure, glucose levels, and respiration rates can provide additional insights into an individual’s health.
- Development of personalized health monitoring systems: The proposed framework is designed to monitor the health of individuals in a general sense. However, personalized health monitoring systems can be developed by using deep learning techniques to analyze an individual’s historical health data and provide customized recommendations for improving their health.
- Enhancing the security and privacy of health data: As more health data is collected and transmitted through IoT devices, ensuring the security and privacy of this data becomes increasingly important. Future research can focus on developing robust security and privacy mechanisms to protect health data from unauthorized access and breaches.
- Integration with telemedicine services: The proposed framework can be further enhanced by integrating it with telemedicine services. This would enable healthcare providers to monitor and diagnose patients remotely, reducing the need for in-person visits and improving access to healthcare in rural and remote areas.
- Evaluation of the impact of home environment health monitoring on healthcare outcomes: While home environment health monitoring has the potential to improve healthcare outcomes, the actual impact of these systems on patient health needs to be evaluated. Future research can focus on conducting clinical studies to evaluate the effectiveness of home environment health monitoring systems in improving healthcare outcomes.

We believe this research will allow further exploration in this field.

## 6. Conclusions

This paper proposes an IoT-based system for remote monitoring and early detection of health issues in home clinical settings. By integrating IoT devices such as the MAX30100 pulse oximeter, AD8232 ECG sensor module, and MLX90614 non-contact infrared body temperature sensor, we collected crucial physiological data, including blood oxygen level, heart rate, body temperature, and ECG signals. The collected data was transmitted to a server using the MQTT protocol and analyzed using a pre-trained deep-learning model based on a convolutional neural network with an attention layer. The proposed system successfully classified five different categories of heartbeats from ECG signals and detected fever or non-fever conditions based on body temperature. It also provided a comprehensive report on the patient’s heart rate and oxygen level, indicating whether they fell within normal ranges. Critical abnormalities were automatically flagged, prompting a connection to the nearest doctor for further diagnosis. The integration of IoT and deep learning technologies showcased the significant potential for remote health monitoring and disease prediction. By leveraging wearable sensors and miniature devices, real-time physiological data can be obtained, while deep learning algorithms enable early detection of health problems. The MQTT protocol ensured efficient and secure data transmission, while the cloud-based architecture facilitated data processing and analysis. Developing deep learning models for health problem classification holds promise for further enhancing accuracy and reducing reliance on manual feature extraction. The combination of IoT and deep learning technologies is poised to revolutionize the healthcare industry, enabling proactive disease prevention, remote monitoring, and early detection of health issues.

## Figures and Tables

**Table 1:** Configurations and Specifications of IoT System Components.

Component	Specifications
NodeMCU	Open-source firmware and development board based on the ESP8266 Wi-Fi module. It has a USB interface, voltage regulator for stable power supply, 11 digital I/O pins, 1 analog input pin, and 1 UART communication interface. Compatible with Arduino IDE.
MAX30100	High-sensitivity pulse oximeter and heart rate sensor module. Measures SpO2 and HR using integrated LEDs, photodetectors, and low-noise electronics. Includes ambient light cancellation algorithm for improved accuracy.
AD8232 ECG sensor	Single-lead ECG sensor with low power consumption and small form factor. Includes an instrumentation amplifier, right-leg drive amplifier, lead-off detection circuit, and comparator. Measures ECG signals accurately and can detect arrhythmias and heart rate.
MLX90614 Temperature Sensor	Non-contact infrared temperature sensor with a wide measurement range of −70 °C to 380 °C. Uses an infrared-sensitive thermopile detector for temperature measurement. Provides calibrated digital output for ambient and object temperatures over an I2C interface.

**Table 2:** Hyperparameter values and their corresponding values used in the grid search for the proposed approach.

Parameter	Value 1	Value 2	Value 3	Value Used
Number of filters	32, 64, 128	64, 128, 256	128, 256, 512	64, 128, 256
Neuron size	128, 256	256, 512	512, 256	512, 256
Dropout rate	0.2	0.5	0.8	0.5
Learning rate	0.1	0.01	0.001	0.001
Batch size	128	256	512	512
Epochs	25	40	50	25

**Table 3:** Classification Report for the proposed model Performance.

Class	Precision	Recall	F1-Score	Support
Normal Beat	0.98	1.00	0.99	18,118
Supraventricular premature beat	0.90	0.78	0.83	556
Premature ventricular contraction	0.97	0.93	0.95	1448
Fusion of ventricular	0.88	0.50	0.64	162
Unclassifiable beat	0.99	0.99	0.99	1608
accuracy			0.98	21,892
macro avg	0.94	0.84	0.88	21,892
weighted avg	0.98	0.98	0.98	21,892

## Data Availability

There is no statement regarding the data.
